# Concepts and definitions of health and health-related values in the knowledge landscapes of the digital society

**DOI:** 10.3325/cmj.2017.58.431

**Published:** 2017-12

**Authors:** Anna Lydia Svalastog, Doncho Donev, Nina Jahren Kristoffersen, Srećko Gajović

**Affiliations:** 1 Østfold University College, Department of Health and Social Studies, Fredrikstad, Norway; 2 University “Ss Cyril and Methodius”, Faculty of Medicine, Institute of Social Medicine, Skopje, Republic of Macedonia; 3 University of Zagreb School of Medicine, Croatian Institute for Brain Research, Zagreb, Croatia *srecko.gajovic@hiim.hr*

The knowledge landscapes (*http://knowledge-landscapes.hiim.hr/*) represent multidimensional environments, which individuals encounter when searching for knowledge, particularly, knowledge related to health (1,2). In digital society, knowledge is well distributed virtually and online. A substantial fraction of knowledge landscapes is also located in the digital environment. Both health and knowledge need to be understood today as a part of the digital society. It is important to estimate the impact that digital society has on knowledge landscapes and on health. This is especially relevant in relation to the 90th anniversary of Zagreb Andrija Štampar School of Public Health and the work of its founder, Andrija Štampar, with ongoing ambition of the School to be on the leading edge of public health understanding in the present society (3). Hence, this article deals with the concepts of health in relation to the present digital environment.

How knowledge relevant to health and well-being is distributed in the digital society, and how the search for this knowledge, ie, navigating knowledge landscapes, influences everyday life and health needs to be clarified. We present different definitions of health and health-related values. The usual approach to explaining health definitions would be to give a historical and chronological overview showing the development of the ideas over time to better understand the current position. In contrast to this, in the online environment, the overload of information and limitless opportunities of content presentation result in co-existence of different views. We claim that, today, we need a historical overview to identify and understand this multiplicity of views and standpoints co-existing in the digital environment. This co-existence being the product of digital society could be referred to as “digital anachronism”.

## Historical overview of attitudes and considerations about health – from concepts of balance to ideas about health as economic category

The concept of health as a balance between a person and the environment, the unity of soul and body, and the natural origin of disease, was the backbone of the perception of health in ancient Greece. Similar concepts existed in ancient Indian and Chinese medicine (4,5). In the 5th century BC, Pindar defined health as “harmonious functioning of the organs”, emphasizing the physical dimension of health, the physical body and the overall functionality, accompanied by the feeling of comfort and absence of pain. Even today, his definition bears importance as a prerequisite for the overall health and wellness. Plato (429-347 BC) in his “Dialogues” pointed out that a perfect human society could be achieved by harmonizing the interests of the individual and the community, and that the ideal of ancient Greek philosophy “a healthy mind in a healthy body” could be achieved if people established internal harmony and harmony with the physical and the social environment. According to Aristotle’s teaching, man is a social being by his very nature; he tends to live in communities with the duty to respect the moral standards and ethical rules. Aristotle emphasized the necessity for regulating the relations in the society to achieve harmonious functioning and preservation of health of its members. Democritus connected health with behavior, wandering why people prayed to God for health, which was essentially under their own control. Hippocrates explained health in connection with the environmental factors and lifestyle. Hippocrates was the creator of the concept of “positive health”, which depended on the primary human constitution (we consider it today as genetics), diet, and exercise. He thought that proper diet and exercise were essential for health, and that seasons’ changes had a profound effect on the mind and body, resulting in different types of predominant diseases during the winter (respiratory tract diseases) and summer (digestive tract diseases) (4,5). A lot might be said about the long standing philosophical discussion about body and soul, and in present society between body and mind, as an active dichotomy (Plato and Hellenism) or as an integrated unity (usually reference to Aristotle), which is important to know about in the current online environment.

In the Middle Ages, health perception was strongly influenced by religion and the church. After Roman Empire fell apart, the church was left as an only important infrastructure providing care for the people and collecting the knowledge on remedies, eg, herbs grown in monastery gardens (6). The “forgotten” knowledge of antiquity was re-discovered during the Renaissance and re-framed up to the present. During the period of Industrial Revolution, health became an economic category, which was to allow good condition and working ability and reduce lost work days due to illness. Accordingly, the value of health was such as enabling economic profit. The health was intertwined with Darwinian understandings of strength and being the fittest, where meaning of life was tied to physical survival. Another health aspect considered the ability of the individual to adapt to the influences from the environment to the extent that the individual could tolerate and resist. When the adjustment is over, the disease occurs as a natural consequence. This approach first reflected only biological mechanisms of adaptation, later adding on influences from the environment, which needed to be governed and modified (4,5).

## Modern concepts of health – from individual to societal risks and back to the individual

All modern concepts of health recognize health as more than the absence of disease, implying a maximum capacity of the individual for self-realization and self-fulfillment. This should equilibrate the human inner forces and possibilities with the feeling of pleasure or dissatisfaction in their relations with the environment (7). Social medicine and public health approach to health advocate that we should not only observe the health of the individuals, but also the health of the groups and the community, as a result of the interaction of the individuals with the social environment.

The holistic concept of health is contained in the expression of wholeness. Health is a relative state in which one is able to function well physically, mentally, socially, and spiritually to express the full range of one’s unique potentialities within the environment in which one lives. Both health and illness are dynamic processes and each person is located on a graduated scale or continuous spectrum (continuum) ranging from wellness and optimal functioning in every aspect of one’s life, at one end, to illness culminating in death, at the other (3,8).

The theory of salutogenesis takes a different view of what creates health and what factors support health, as opposed to the conventional approach of pathogenesis to study the factors that cause disease (9). To find the “origins of health”, one needs to search for factors that support the human health and welfare (10).

## Definitions of health by the World Health Organization

To establish social welfare and to facilitate, encourage, and secure individual autonomy and dignity are key challenges in the present time and society. The modern understanding of health became official when the World Health Organization (WHO), at the time of its establishment in 1948, included the definition of health in its Constitution. The definition was proposed by Dr Andrija Štampar, a prominent scholar from Croatia in the field of social medicine and public health and one of the founders of the WHO. This generally accepted definition states that “health is a state of complete physical, mental, and social well-being and not merely the absence of disease or infirmity” (11). This definition promoted for the first time that, in addition to physical and mental health, social welfare is an integral component of the overall health, because health is closely linked to the social environment and living and working conditions.

Respecting this definition as a global concept, many researchers and theorists subsequently advocated for adoption of working, practical, and operational definitions of health. In 1977, with the adoption of the WHO Global Strategy “Health for All by the Year 2000”, a pragmatic concept of health – the ability to conduct a socially and economically productive life – was accepted indirectly, which was an essential goal of this Strategy (12).

To define health in operational and working terms was vital for creating policies and programs for maintaining and improving health, and it considerably managed to exceed the widely rooted notion that health simply means the absence of disease. The Ottawa Charter from the 1st International Conference on Health Promotion, held in Ottawa, Canada, in 1986, says that health is created in the context of everyday life and environment, where people live, love, work, and play. Thus, active and interactive understanding of health was introduced. The goal of health promotion is to combine the approach for addressing the social determinants with the resolution and commitment to motivate and encourage the individuals and the community for their active approach toward health and embracing healthy lifestyles (13-16).

Within the last few decades, the WHO definition of health has been increasingly amended and supplemented by the fourth dimension – spiritual health. Generally speaking, spiritual health involves a sense of fulfillment and satisfaction with our own lives, system of values, self-confidence and self-esteem, self-awareness and presence, peacefulness and tranquility with dynamic emotional balance, both internal and toward the environment, morality and truthfulness, selflessness, positive emotions, compassion and willingness to help and support others, responsibility and contribution to the common good, and successful management of everyday life problems and demands as well as social stress (17).

## How the health is perceived in the current digital environment – a lay person’s perspective

The digital society, where information technology has led to fundamental societal shifts, is a carrier of particular characteristics. It got new organizing principles (relations and arenas) for public institutions and private agents, and it has also altered the relation between public and private (18). In digital society, information technology has become something more than a tool for communication, storage, and sharing of information. Therefore, it should not be reduced to another story we add to the former stories of society. Instead, the society has altered as such, and it needs to be described on its own terms.

To act as a citizen in a digital society presupposes having particular skills on how to get hold on knowledge and how to access, interpret, and use it in the online environment. In the digital society, knowledge is organized in dynamic tentative online infrastructures and made available to users through different search tools and engines and their operative algorithms. This means that digital society frames, alters, and produces knowledge in a complex way, and the individuals today need skills to read these continuously changing landscapes critically and navigate them safely (1,2,19,20). We have conceptualized this multidimensional, technology-enabled environment, open to individual access, as the knowledge landscapes (1). As a citizen, the individual needs to know how to navigate these knowledge landscapes to gain health-related information and be able to decide on strategies and services for one’s own life and the lives of others one cares for.

The wide array of producers contribute to the contents to be found online, which enables them to express their own thoughts and visions, in particular in regard to health. An important question is whether societally prescribed values and theoretical positions are also reflected in lay people’s empirical understandings and perceptions of health. Health professionals and politicians also need to know what people themselves perceive as the most important issues regarding health, in particular what health is, and which factors in people’s lives constitute health. This knowledge is needed to meet individuals in various health care settings and to deal with health issues across the digital realm.

Research on individual perceptions regarding health and illness has been accumulating for some time, and it is becoming apparent that they have significant consequences on the person’s health behavior (21). It is found that gender and age influence people’s perceptions of health as much as their background and environmental factors. Overall, lay people’s perspective on health and illness should not be viewed as constructs on opposite ends of a single continuum, but rather as two distinct but overlapping constructs (22). Experiences of health are more intangible and elusive than experiences of illness, making the former much more challenging to study (23). Health could be taken for granted and not brought to the persons attention before it’s challenged in situations characterized mainly by the actual or threat of change, disease, or loss.

Zahra et al (24) studied lay people’s perceptions of health and factors affecting health across 29 countries. People belonging to different backgrounds had different perceptions regarding determinants of health. The highest percentage of people agreed that environment was the determinant of health, which was consistent with the scientific view of increased burden of diseases caused by environmental factors. Fugelli & Ingstad (25,26) conducted a multi-sited ethnographic study to explore lay people’s perceptions of health in different contexts, environments, and sites. They interviewed people in their own homes, in five different locations in Norway, in rural areas, small and big cities, people from different socioeconomic and cultural background, living on the coast, inland, in fishing-, agriculture- and industrial communities. They identified six essential elements in people’s conceptualization of health in their actual situations: well-being, function, nature, a sense of humor, coping, and having energy.

The lay perspective on health appears to be characterized by three qualities: wholeness, pragmatism, and individualism. *Wholeness* is related to health as a *holistic phenomenon.* Health is an aspect interwoven with all other aspects of life, everyday life, working life, family life, and community life. Health is viewed a resource and a total, personal, situation-specific phenomenon. Absence of disease is not enough – the life situation as a whole must be taken into consideration. Family functioning and children’s welfare is an important part of experiencing health as wholeness. To be able to live according to one’s personal values is also an important issue. *Pragmatism* reflects the health as a *relative phenomenon*. Health is experienced and evaluated according to what people find reasonable to expect, given their age, medical conditions, and social situation. In this way, health is not necessarily freedom from disease or loss of functional abilities. Other positive values in life can compensate for different types of losses. Most people are realistic in their life-expectations. Finally, *individualism* relates to health as a highly *personal phenomenon*. The perception of health depends on who you are as a person. To be part of a society and to feel close to some other persons seems to be important to all. Furthermore, values are individual and, as every human being is unique, strategies for improving health must be individualized.

## Health definitions at the intersection of technology, medicine, and individuals in the digital society

The digital society allows different perspectives to co-exist and dynamically evolve in the different forms of online environment. Subsequently, the different views on health are present online in the same time, competing for attention of the visitors, users, and creators of the digital content. Some of these ideas, although seemingly new and appealing, frequently represent refurbished historical concepts. Moreover, every public health-related intervention should consider its online context. To be able to identify and recognize the individual understanding of health is important, in particular when this conception of health contradicts recommendations for diseases that need medical intervention.

The plurality of health definitions reflects the variety of contexts in which health is elaborated. A concept ‘home context’ is the context in which the concept originates. It makes sense predominantly inside but not necessarily outside its home context. Conceptualizing is a verbal act that sets out to identify and shape phenomena’s border and content. The conceptualization localizes and attributes identity, content, or meaning. The definitions of health obviously reflect socially and culturally constructed and tentative categories. The relation between an individual and society is implied in most concepts of health. In particular, in the digital environment, critical analysis of health concepts helps us to understand better health policies and politics and their consequences. As they frequently represent societal powers rather than phenomenological differences, they are accordingly accepted, criticized, or even rejected.

Today’s society is a complex, high-cost, high-tech society where citizens constantly need to learn and update their knowledge and skills to be able to manage their own lives. As digital society is built on software that is constantly renewed or replaced by new software, navigation has become a new ‘skill of hunting and gathering’. To appreciate concepts and conceptualizations, knowledge has become a key quality in digital society. The health concepts – particularly concepts we use to explain, treat, heal or cope with disease – are words we also use as search tools. A feature of digital society is that past and present categories and understandings are available at the same time, undermining the traditional western schooling system, where accumulation of past knowledge is understood and acknowledged as predecessor to present, updated knowledge. We refer to this syncreticity, co-existence of different views without hierarchy and without precedence, as “digital anachronism”. Subsequently, public information and public interventions in regard to health should consider this variety of approaches online and thus themselves be explicit and also argue for why and how a particular approach to health is chosen. The definitions of health presented here are also understood as *navigation-tools* to be applied within online environment. Being aware of them can help the navigator to understand and interpret the information, texts, or documents that occur in online encounters of the health related issues.
